# Phylogeography of the dugong (*Dugong dugon*) based on historical samples identifies vulnerable Indian Ocean populations

**DOI:** 10.1371/journal.pone.0219350

**Published:** 2019-09-11

**Authors:** Stephanie Plön, Vibha Thakur, Leslee Parr, Shane D. Lavery

**Affiliations:** 1 African Earth Observation Network (AEON)- Earth Stewardship Science Research Institute (ESSRI), Nelson Mandela University, Port Elizabeth, South Africa; 2 School of Biological Sciences, University of Auckland, Auckland, New Zealand; 3 Department of Biological Sciences, San José State University, San José, California, United States of America; 4 Institute of Marine Science, University of Auckland, Auckland, New Zealand; National Cheng Kung University, TAIWAN

## Abstract

We investigated the phylogeography of the dugong (*Dugong dugon*) across its original range using museum material from 14 natural history museum and university collections. The mitochondrial DNA control region was successfully amplified from samples of bone or tooth powder from 162 individuals. These samples range from 1827 to 1996 and span the historical distribution range of the dugong. We were able to successfully amplify overlapping fragments of the D-loop region of the mitochondrial DNA (mtDNA) resulting in sequences of a 355 bp fragment for 162 individuals for the final analyses. This included a new sequence (189 bp) from a previously unidentified piece of skin of the extinct Steller’s sea cow (*Hydrodamalis gigas*), as an outgroup. The resulting dugong sequences match those from previous studies of dugongs from Australia and Indonesia, but revealed several new and divergent mtDNA lineages in the Indian Ocean. One mtDNA lineage includes most specimens from the Western Indian Ocean, with another distinct lineage isolated to nearby Madagascar and Comores. There is little geographic structuring detectable among other populations in the Western Indian Ocean and all populations from that region appear to have historically contained comparatively low levels of genetic diversity. The genetic diversity of several Indian Ocean samples collected after 1950 was lower than that of the samples collected earlier from similar locations, a result coincident with the anecdotal reductions in population size. The new lineages and potential loss of diversity highlight the particular conservation importance and vulnerability of dugong populations in the Western Indian Ocean.

## Introduction

The dugong (*Dugong dugon*) is one of four extant species of sea cow (Sirenians). It has the largest extent of occurrence of any Sirenian, extending over some 128,000km of coastline across 37–44 countries in the Indo-Pacific region, including tropical and subtropical coastal and island waters from east Africa to Vanuatu [1, 2]. However, due to the coastal and riverine habitat of sea cows, the long-term survival of all four extant species is in peril due to many confounding factors, the majority of which are the direct or indirect result of human activity [3]. Anecdotal evidence suggests that the dugongs’ area of occupancy has generally declined, particularly along the coast of East Africa and India. The dugong is currently classified as ‘vulnerable’ by the International Union for the Conservation of Nature (IUCN), but its conservation status is highly variable throughout its range and the species may in fact be ‘endangered’ or ‘critically endangered’ in some parts of its range [1, 2, 4].

Historically, dugongs occurred in almost all coastal areas of the Indo-Pacific, from southern Mozambique in the west to Australia and New Caledonia in the east, including many of the Island States in the Indian and Pacific Oceans [2]. Although robust estimates of population size are not available for much of their range, anecdotal evidence suggests that many dugong populations appear to have suffered a steep decline in numbers since the 1960s [2, 5]; many populations are believed now to be small and, where they still do occur, are represented by relict populations only [2, 6]. The larger populations remaining today appear to be off Mozambique, in the Arabian/Persian Gulf and the Red Sea, in several Australian locations, and off New Caledonia, and there is evidence for a reduction of the species' area of occupancy within its range [1, 2, 4,7, 8, 9, 10].

These data suggest that one of the most critical effects, along with habitat fragmentation and a reduction in animal numbers, is a possible loss of genetic diversity. Genetic diversity is the fundamental level of biodiversity [11] and thus a critical first step in developing management programs for threatened species is the determination of existing genetic variability within and between management units [3, 11]. The accurate delineation of distinct management units and the ability to detect changes in genetic diversity within these management units is important for successful conservation planning and management [3].

The dugong’s tendency to use turbid environments and lack of a dorsal fin for individual identification make it even more challenging to study than most other marine mammals using standard observational methods [2, 12]. Consequently, very little is known about dugong population structure and migration patterns. Most movements appear to be short-distance, and although a few studies have shown that individuals can move long distances of up to 1000 km, these are likely vagrants [12, 13]. To date, genetic research on dugong populations has focused primarily on the Australian region and preliminary data suggest substantial population structure in the dugong across the Australian part of its range, and indicate that Australian dugongs are distinct from those in other regions of the Indo-West Pacific [14, 15]. Previous work on the molecular analysis of mitochondrial DNA (mtDNA) sequences from 177 dugongs from the Australian coast using the control region (D-loop) locus identified two divergent mitochondrial lineages, one restricted lineage found primarily off the north-eastern coastline, and a widespread lineage that included specimens from all Australian locations [15, 16]. By contrast, the analysis of a number of microsatellite loci indicated relatively high gene flow across northern Australia [16]. These observations could be the result of migrations occurring primarily in males, with females displaying more site fidelity, which can localize genetic variation in the maternally inherited mitochondrial marker over time. Such a sex-bias in dispersal has been shown on a smaller scale in southern Queensland [17, 18]. Additional research on samples from Thailand showed that those animals are distinct from Australian dugongs ([19]; J. Bushell, unpublished data). Although to date no molecular analysis has been published on dugongs from the Indian Ocean, previous results from studies conducted on samples from Australia and Thailand [12, 15, 19, 20] suggest long-term isolation between dugong maternal lineages and subsequent partial geographic mixing of dugong matrilines.

To date it is unclear exactly how many dugongs remain in the Western Indian Ocean (WIO, here defined as including regions from Mozambique to the Arabian/Persian Gulf), but it appears that the only viable populations are located in the Bazaruto Archipelago off Mozambique [6], off Qatar, Saudi Arabia, and the United Arab Emirates in the Arabian/Persian Gulf [7], as well as the Red Sea [4]. The few remaining animals present in other parts of the WIO are difficult to find and are only rarely sighted [2]. Thus contemporary tissue samples for genetic analysis are extremely difficult to obtain in most localities due to the scarcity, low population numbers, and relative inaccessibility of animals. However, data on population genetic structuring are vital in order to assess the isolation and vulnerability of existing populations and design effective management and conservation strategies if necessary. Thus in an effort to source alternative samples for DNA analysis to determine population structuring of the dugong throughout its range, we employed molecular techniques using samples from museum specimens originating from the region and obtained under former colonial rule. A population genetic analysis of this material should permit the identification of important management units, which may require special protection and conservation, thus such data can assist in effective management and conservation planning for this vulnerable species.

The extraction of mitochondrial DNA (mtDNA) from tissues containing only low levels of genetic material, such as hair, faeces, eggshells, teeth and bone (also often referred to as ancient DNA or aDNA analysis [21, 22] has become a commonly-used tool in conservation genetics in the last two decades or so [23, 24, 25, 26, 27, 28, 29, 30], and has been particularly successful for a number of marine mammal species that are inaccessible in the wild [31, 32, 33, 34, 35, 36]. In a few cases the use of this technique has even resulted in the discovery of new, or the resurrection of previously described, marine mammal species [37, 38]. The importance of museum material in these studies has been highlighted [39, 40]. This tool is particularly powerful in cases where animals in the wild are either inaccessible [4, 35] or where too few specimens are left to warrant sampling of genetic material from wild animals, for which this may be deemed too invasive and stressful for the animals, as in the case of the dugong (Nick Gales, *pers comm*.). Since genetic analysis can provide information that is not available by any other means, the analysis of aDNA samples of dugong throughout its historical range can assist in determining genetically isolated populations and thus aid in delineating management units.

Ideally, an assessment of the genetic distinction of populations, delineation of management units, and evaluation of gene flow between them is carried out as a conservation tool before a species becomes vulnerable or even endangered [41, 42]. However, increasing numbers of large mammal species are facing rapid population declines without this detailed knowledge being available. As a result, museum collections are being used increasingly to aid in the retrospective assessment of historic genetic population structuring in an effort to help conserve and manage contemporary, now relict populations and assist in their recovery. Thus in the absence of contemporary samples, particularly from the Western Indian Ocean region, we obtained and analysed historical skeletal museum samples (bones and teeth/tusks) of dugong *Dugong dugon*. We also obtained a historical sample (dried skin) assumed to be from the extinct Steller’s sea cow (*Hydrodamalis gigas*) to provide a closely related outgroup. The date of acquisition or geographical location for this sample was unknown; however, given the description of the species in 1741 and subsequent rapid extinction a few decades later, the age of the sample is likely around 240 to 280 years old.

The aim of the present study was to determine the genetic diversity and phylogeographic structuring of the dugong throughout its historical range based on these museum samples, in order to identify historically significant management units for effective conservation planning and implementation. Exhaustive comparisons with all known existing dugong mtDNA sequences await the imminent publication by other research groups of several additional mtDNA data sets from newly-sampled regions in the West Pacific.

## Materials and methods

### Sample collection/DNA extraction

**Fig 1:**
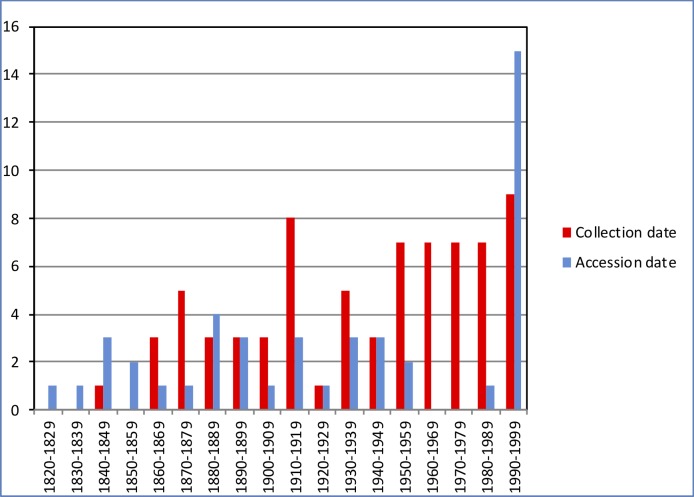
Sample distribution over time. Collection date (grey) and accession date (black), respectively, are shown for 119 samples for which this information was available and for which sequences could be obtained.

**Fig 2:**
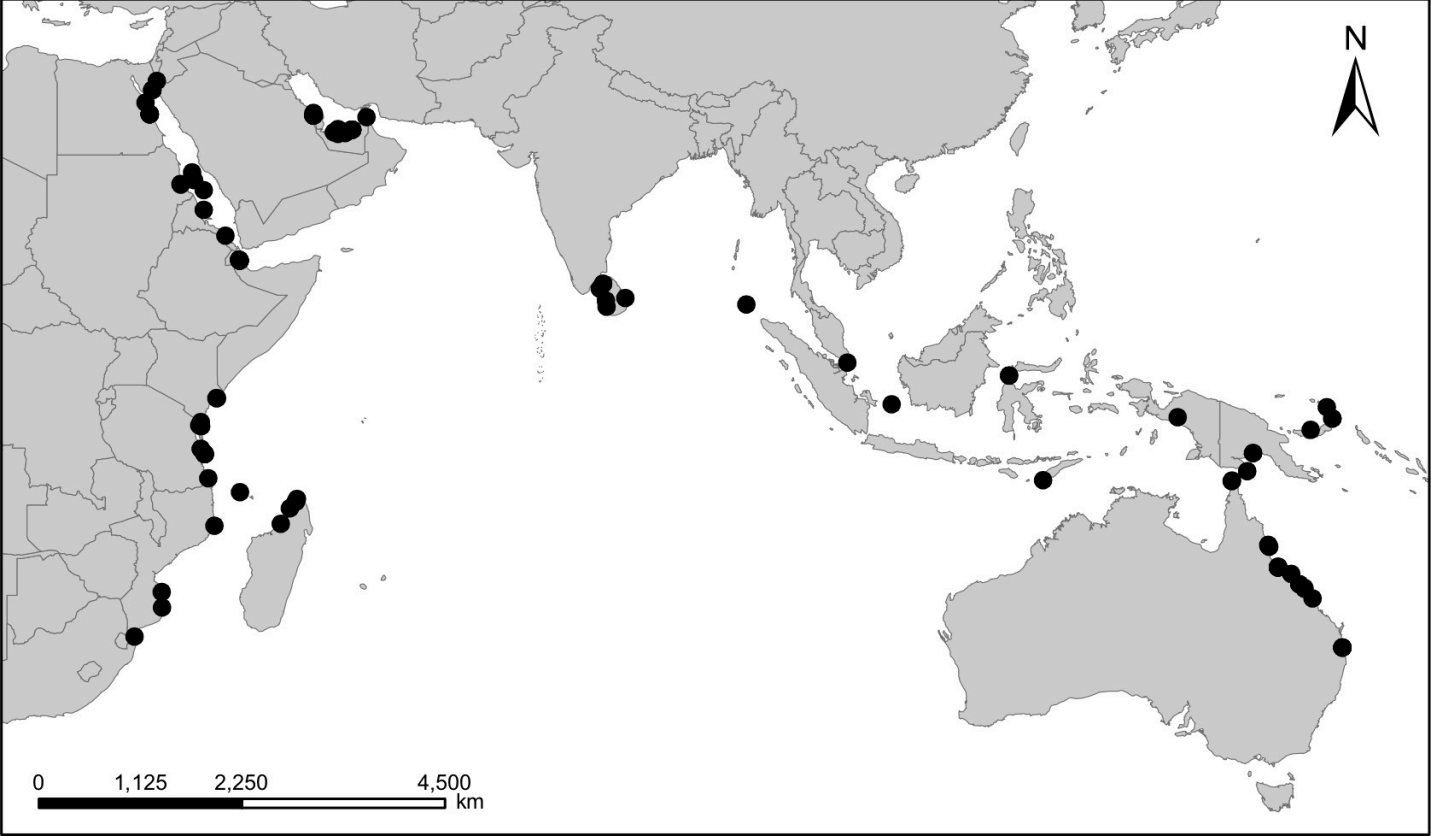
Geographic origins of museum samples. Each dot could represent one or more specimens per locality.

Samples of bone and tooth powder were obtained from a total of 177 individual dugongs located in 14 collections (11 European and one African museum collection, and two European university collections; Table 1). All samples were obtained on loan from the individual collections (see S3 Table) and covered by individual CITES (Convention for the International Trade in Endangered Species) permits. Available catalogue information included collection date and/or accession date. Where collection date was not available, accession date was used as the approximate collection date for that sampled individual. For 119 individuals from which sequences could be obtained and for which date information was available, the dates ranged from 1827 to 1996 (Fig 1). The 115 individuals from which sequences could be obtained and that had geographical location associated with them spanned the entire historical distribution range of the dugong (Fig 2).

**Table 1:** Breakdown of samples by museum collection.

Museum collection	No. of individual specimens sampled	No. of individual specimens from which sequences were obtained	No. of sequenced individuals for which geographical information was available	No. of sequenced individuals for which accession and/or collection date was known
London (UK)-Natural History Museum	62	58	47	50
Edinburgh (UK)-National Museums of Scotland	17	17	8	16
Leiden (NL)-Naturalis	15	14	9	7
Bremen (D)-Überseemuseum	15	13	3	3
Stuttgart (D)-Staatliches Museum für Naturkunde	12	12	10	9
Paris (F)-Musee D’Histoire Naturelle	12	12	11	12
München (D)-Zoologische Staatssammlung	10	9	7	8
Port Elizabeth (ZA)- Port Elizabeth Museum	8	8	7	6
Vienna (A)-Naturhistorisches Museum	8	7	5	6
Berlin (D)-Museum für Naturkunde	6	5	5	0
Prague (Cz)-National Museum	6	4	1	0
Dresden (D)-Senckenberg Naturhistorische Sammlungen	2	0	0	0
University Zoological Collections (D)- Hamburg, Rostock	4	3	2	2
**Total**	**177**	**162**	**115**	**119**

Sampling of bone and tooth/tusk material was carried out with a hand-held drill and a 1.5mm drill bit. For the purpose of aDNA analysis, 0.05g (50mg) of bone or tooth powder was sufficient for extraction. A new 1.5mm drillbit was used for each sample and an effort was made not to drill more than 5 or 6 samples in the same location to avoid cross-contamination between samples. Drilling was restricted to skulls and teeth/tusks only, to prevent accidental resampling of the same specimen. The evacuation and relocation of European collections during World War II may have resulted in skulls being separated from the remainder of the skeleton, thus sampling of both skulls and skeletons could potentially have resulted in unknowingly sampling the same individual specimen twice.

In addition, for use as an outgroup, a sample was taken from a putative piece of skin from a Steller’s sea cow (*H*. *gigas*), which was detected in the collection of the Überseemuseum in Bremen, Germany. The sampling method was the same as for dugong samples.

The primary laboratory analysis was conducted in a laboratory isolated from any mammal DNA in the School of Biological Sciences, University of Auckland, New Zealand. Only equipment and reagents dedicated for the sole purpose of this project were used.

Total genomic DNA was isolated from powdered bone and tooth samples using bone extraction buffer [42] and purified using a DNeasy Blood & Tissue kit (Qiagen Inc., Valencia, CA, USA), following the manufacturer’s protocols.

A brief protocol to ensure quality control has been included in the supplementary materials (S1 Protocol).

### Control region primer design and PCR amplification

Four PCR primers previously designed for modern samples (DugCR_5F and DugCR_369R [42]; DugDLF and DugDLR [15]) were initially used for amplification, but did not yield the expected products. As aDNA may often be fragmented, depending on the state of preservation, six custom-designed primers were then used to obtain overlapping as well as forward and reverse sequences for a 355bp long region of the d-loop of the mtDNA of the dugong:

Primers:

DugCR_5F 5’-CTA CTT AAA CTA CTC CCT GCG-3’ [42]
DugCR_369R 5’-CGG AGC GGG TTG CTG GTT TCT-3’ [42]
DugDLF 5’-CAT ATT ACA ACG GTC TTG TAA ACC-3’ [15]
DugDLR 5’-GTC ATA AGT CCA TCG AGA TGT C-3’ [15]
DugCR 3F 5’-TTC TAC TTA AAC TAC TCC CTG CGC-3’ (present study)
DugCR 93F 5’- ACA CCA TCC TAT GTA TAA TCG TGC A-3’ (present study)
DugCR 114F 5’- TGC ATT ACA CTA CTT ACC CCA TGC-3’ (present study)
DugCR 137R 5’- GCA TGG GGT AAG TAG TGT AAT GCA-3’ (present study)
DugCR 322R 5’- ATT GGA GGT GAT AAG CGT GTT GA-3’ (present study)
DugCR 359R 5’- GCT GGT TTC TCG AAG CTT GGT A-3’ (present study)

PCR reactions were conducted in 25 μl reaction volumes, using Platinum *Taq* DNA polymerase (*Invitrogen*) and 10x reaction buffer. Reactions generally contained 2 mM MgCl2, 0.4 mg/ml bovine serum albumin (BSA), 0.25 mM dNTPs, 0.4 μM forward and reverse primers, and 2μl of DNA template. The PCR conditions were as follows: 95°C for 5 min, 50 cycles of (95°C for 30 s, 55°C for 30 s, 72°C for 30 s), and 72°C for 10 min.

PCR products were purified using Exonuclease I and Shrimp Alkaline Phosphatase [43], sequenced (in both directions using the amplification primers) with the BigDye Terminator Cycle Sequencing Ready Reaction kit (Applied Biosystems) and separated on an ABI 3130xl DNA Sequencer (Applied Biosystems) at the School of Biological Sciences (SBS), University of Auckland, New Zealand. Sequences were edited and aligned manually (and with the Muscle algorithm where necessary) using the program GENEIOUS v. 7.0.6. [44]. (GenBank accession numbers: MH704268-MH704430; Steller’s sea cow *Hydrodamalis gigas*: MH 717817). Several other sequences purportedly from Steller’s sea cow mitochondrial control region exist on GenBank (acc. nos. KP134338-42), but do not align well with our sequence from this species, or with the other sirenian control region sequences, and therefore we believe these to be inaccurate.

### Analyses

Phylogenetic tree construction was undertaken on the alignment of sequences using the program GENEIOUS v. 7.0.6. [44]. We employed a combination of neighbour-joining, maximum likelihood and Bayesian analyses. Previously defined mtDNA lineages were identified by alignment and monophyletic clustering with all reference sequences from each lineage. A median-joining network of relationships among the mtDNA haplotypes was constructed using Network [45]. New, previously undescribed mtDNA lineages were defined as distinct haplogroups (monophyletic groups of related haplotypes) that differed from existing lineages by more than two base changes.

An Analysis of Molecular Variance (AMOVA) was carried out in ARLEQUIN v.3.5.1.3 [46] to examine population structure. For this analysis the samples were pooled into the following previously recognised biogeographical regions [47]: 1- Madagascar/Comores, 2- East Africa, 3- Red Sea, 4- Arabian/Persian Gulf, 5- Sri Lanka, 6- Indonesia/N. Australia, 7- East Australia. Both ϕ_ST_ and F_ST_ measures were calculated. The effect of multiple simultaneous pairwise tests was accounted for through FDR (false discovery rate) correction [48].

Estimation of the dates of dugong lineage divergence were undertaken using Beast 2.4.4 [49], based on a 355bp alignment of dugong and outgroups. The Steller’s sea cow had a shorter 189bp sequence, but this analysis makes use of all data and does not delete characters that are missing some data. The tree obtained without the Steller’s sea cow sequence is consistent with the tree including it, but the confidence intervals of divergence dates are smaller when the Steller’s sea cow is included. The best evolutionary model was estimated using bModelTest [50], which inferred model”123121”, a derivation of the HKY model, to have the highest posterior support. A relaxed log normal clock model was used for the tree. MRCA priors were set for all known species divergences in the Sirenian lineage and outgroup, based on previous fossil, morphological and molecular analysis [42, 51] (*Hydrodamalis* + *Dugong* 28.6MYA (28.1–29.9), Sirenia 41.6 (41.3–42.2), Proboscidea 7.2 (6.8–8.1), Tethytheria 65.0 (63.9–65.8)). An MCMC chain length of 100 million generations was used, with sampling every 1000 generations and 10% burn-in. To ensure adequate mixing, effective sample sizes (ESS) greater than 200 were maintained for all model parameters. Convergence was ensured by using multiple independent runs. A maximum clade credibility tree was constructed from the resulting Bayesian trees using TreeAnnotator 2.4.4 and FigTree 1.4.2. The same analysis parameters were used in Extended Bayesian Skyline Plot analyses [52], to estimate change in population size over time for each of the major mtDNA lineages. The 95% HPD (highest posterior density) distribution of the inferred number of population changes was used to determine if the null hypothesis of constant population size could be rejected.

A range of measures of genetic diversity and departure from neutrality were calculated using ARLEQUIN v.3.5.1.3 [46] and GenAlEx 6.5 [53]. Those specimens for which there were dates of collection or accession were divided by time into two approximately equal size groups (before 1950 and after 1950) for a balanced comparison. Diversity measures (and their standard errors) were then calculated for each of these two groups both overall, and for each population or region.

## Results

### Sequence assembly

In total, 533 sequences were produced from a 355 bp fragment of the mt D-loop (beginning at position three relative to the sequences from [15], which contains the region of highest variation in this locus (67 variable sites). Of these sequences, 502 proved sufficiently reliable to be included in the final analyses and originated from 162 individuals (for provenance of samples please see S3 Table). A contig of overlapping sequences was aligned for each individual, each sequence originating from a separate PCR reaction to ensure that sequence artefacts were not included. A number of sequences were also produced from replicate DNA extractions from the same individual to help ensure that sequence artefacts were not included. All replicate samples yielded identical sequencing results. Each contig for each individual used in the analyses comprised between 2 and 6 individual sequences (mode: 4), each produced from separate PCR products, sequenced in forward and reverse directions. The remaining individuals either did not produce PCR products for sequencing or did not produce sequences of sufficient quality or length for inclusion in the analyses.

Multiple sequences obtained from multiple PCR products were identical for each individual, indicating no evidence of artefacts or contamination. In addition, all sequences of known geographic origin were similar to existing reference sequences from the same geographic region where available [15], thus all sequences were deemed geographically reliable.

**Fig 3:**
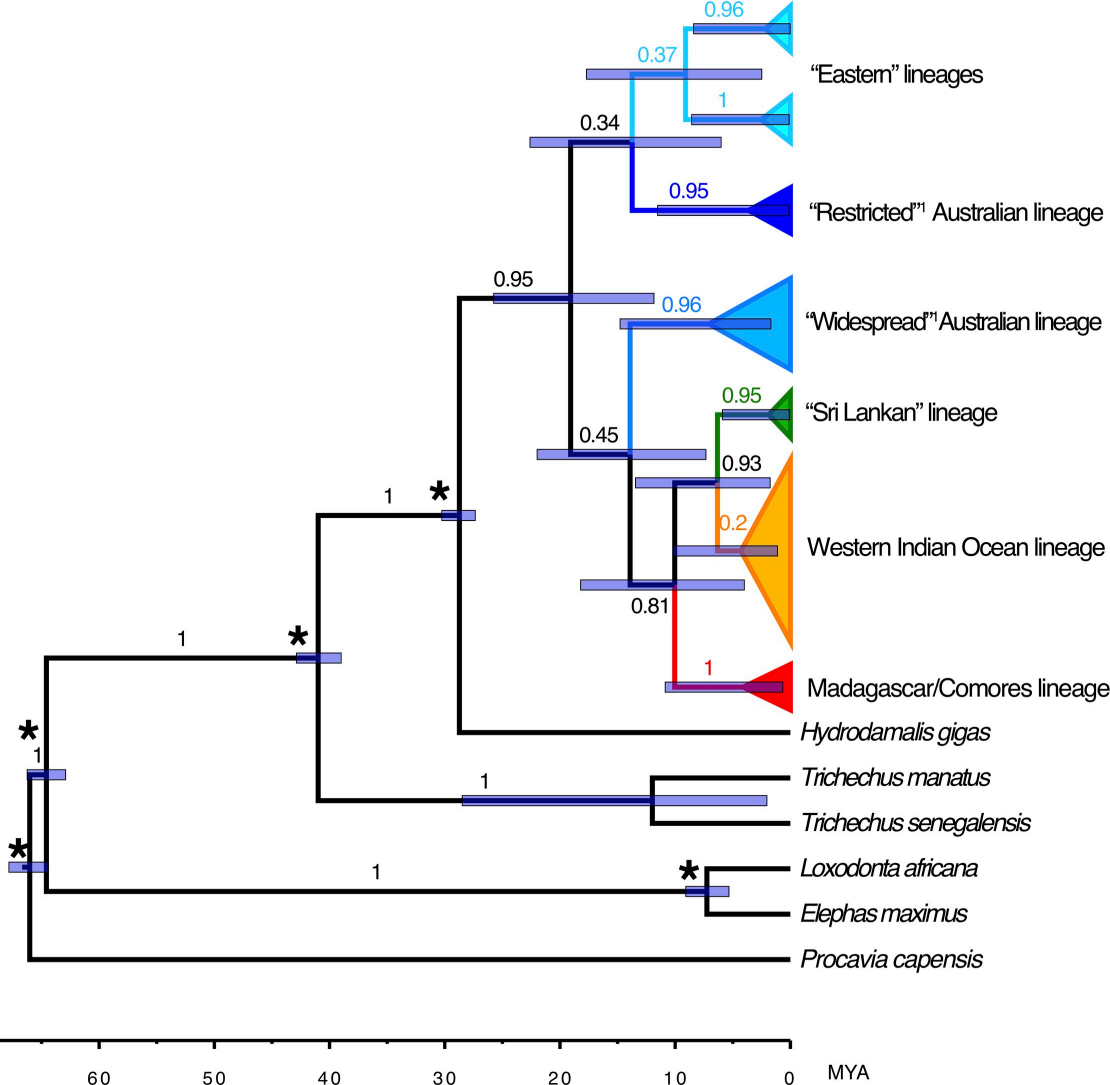
Maximum clade credibility tree for dugong mtDNA haplotypes, rooted with hyrax and elephants, showing estimated ages of clade MRCAs (most recent common ancestors). Compiled from Bayesian MCMC analyses implemented in Beast. Bayesian clade posterior probabilities are indicated on nodes. Node ages are presented as median node heights with 95% HPD intervals represented by bars. Asterisks indicate nodes given age priors (from Springer et al 2015). Lineage names are defined in Fig 4.

In addition, we were able to produce a 189 bp mtDNA control region sequence from the putative piece of skin from a Steller’s sea cow (*H*. *gigas*). This specimen came from the Überseemuseum in Bremen, Germany, and had been previously unidentified, but was suspected to be Steller's sea cow skin; no further information regarding the geographical origin or date of the sample was available. Phylogenetic analyses (Fig 3, plus additional, unreported neighbor-joining, maximum likelihood and Bayesian analyses) indicated that it came from a distinct species, more closely related to dugongs than manatees, and was thus likely to have truly come from a *H*. *gigas* specimen.

### Phylogeographic analyses

**Fig 4:**
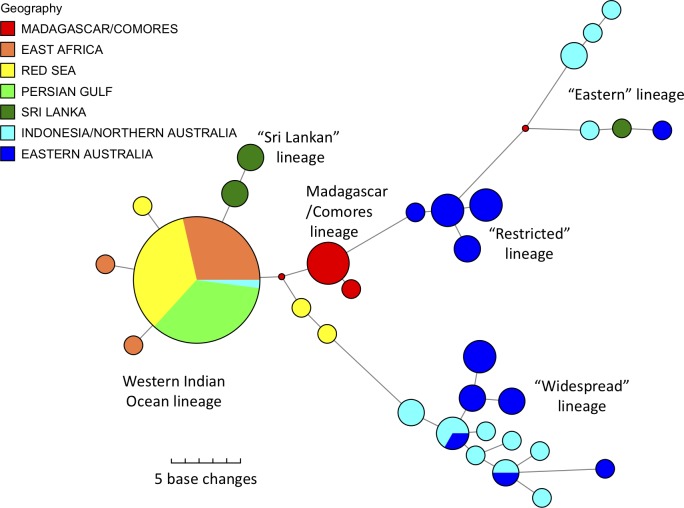
Median-joining network of relationships among dugong mtDNA haplotypes with known location (355bp fragment). Size of coloured pies represent relative frequency. Lengths of joining lines indicate number of base changes. Colours represent regions of origin. “Restricted”, “widespread” and “eastern” lineages previously reported in Blair et al. 2014. The “eastern” lineage refers to the unnamed outgroup lineage of haplotypes in Blair et al. 2014, found in dugong from other parts of the western Pacific beyond Australia. “Sri Lankan”, “WIO” and “Madagascar” lineages first reported here. Small dots represent unsampled median haplotypes.

**Fig 5:**
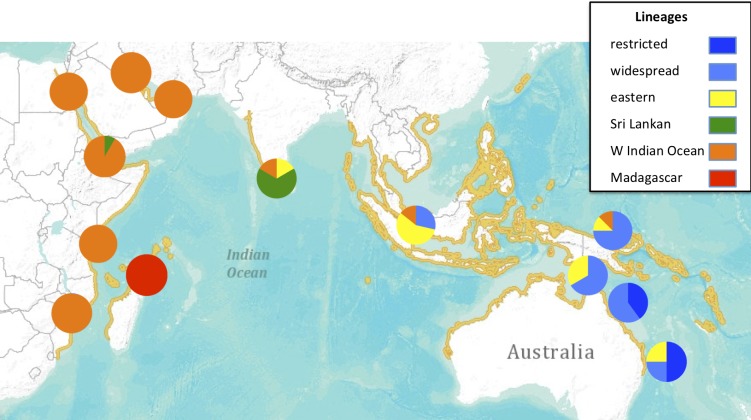
Geographic distribution of main mtDNA lineages for the dugong. Lineage names are defined in Fig 4 and S1 Fig. Reported distribution shown in yellow.

The phylogenetic and network analyses indicated considerable variation among the sequences and showed significant geographic structuring (Fig 4 and S1 Fig). Several previously unreported mtDNA lineages were found among Indian Ocean samples. One new maternal lineage comprised all individuals from Madagascar and the Comores, which were significantly divergent from other, geographically close samples from East Africa. All remaining individuals from the Western Indian Ocean (WIO: including samples from East Africa, Red Sea, Arabian/Persian Gulf) belonged to another distinct, previously unreported lineage. Excluding Madagascar, there appears to be surprisingly little variation among most individuals from throughout the WIO, compared to the variation seen within the eastern range of the species (Figs 4 and 5). However, further sequencing of additional, longer fragments is likely to detect additional variation among the WIO samples. In addition, the distribution and frequency of the major mtDNA lineages indicated that most individuals from Sri Lanka belonged to a slightly divergent lineage and that this regional population appears to be isolated (Figs 4 and 5).

It is particularly noteworthy that the Madagascan/Comores population appears to be the most genetically discrete population so far described in the entire distribution of the species. This is because all individuals sampled from Madagascar and the Comores to date belong to one unique mtDNA lineage. This is unlike any other dugong population, which either share the same lineage as other distant populations (as in the remainder of the WIO), or are distinguished genetically from other populations only by differing frequencies of mtDNA lineages (as seen among the eastern populations) (Figs 4 and 5).

The Analysis of Molecular Variance (AMOVA) indicated an overall F_ST_ of 0.57 and φ_ST_ of 0.51 (both P<0.0001). All geographic regions are significantly differentiated from each other (F_ST_ with P<0.01), with the exception of East Africa, Red Sea, and the Arabian/ Persian Gulf, which show no significant difference from each other. The highest pairwise F_ST_ is found between Madagascar and any of the other regions, ranging from 0.59 to 0.93 (Table 2).

**Table 2:** Pairwise F_ST_ (below diagonal) and φ_ST_ (above diagonal) values of genetic divergence between seven geographic regions of dugongs sampled.

	Madagascar /Comores	East Africa	Red Sea	Arabian/Persian Gulf	Sri Lanka	Indonesia /Northern Australia	East Australia
Madagascar		0.900*	0.822*	0.962*	0.543*	0.520*	0.430*
East Africa	0.839*		0.019	0.004	0.413*	0.607*	0.538*
Red Sea	0.928*	0.019		0.024	0.379*	0.612*	0.547*
Arabian/ Persian Gulf	0.904*	-0.050	-0.024		0.477*	0.623*	0.553*
Sri Lanka	0.700*	0.598*	0.724*	0.662*		0.385*	0.325*
Indon./NAus	0.588*	0.504*	0.632*	0.556*	0.351*		0.110*
E. Australia	0.602*	0.570*	0.681*	0.615*	0.412*	**0.149**	

(Bold = P<0.05, * = significant at .05 level after adjustment for multiple simultaneous tests)

### Lineage divergence dating

The dugong mtDNA control region sequences were aligned with Genbank reference sequences from all closely related species in order to estimate approximate times of divergence between lineages, based on known calibrated fossil divergence dates [54]. Bayesian analyses resulted in estimates of divergence times for each lineage (Fig 3 and S1 Table). These ranged from the most recent divergence of the “Sri Lankan” lineage at approximately 1.9 million years ago (MYA) (95% highest posterior density confidence interval 0.05–5.90), and the Madagascar lineage at 4.1MYA (0.66–10.87), to the coalescence of all dugong lineages at approximately 19MYA (11.84–25.75). It is notable that the divergences among dugong lineages are of the same order of magnitude as those between manatee species and between elephant species (Fig 3).

### Genetic diversity and population demography

**Fig 6:**
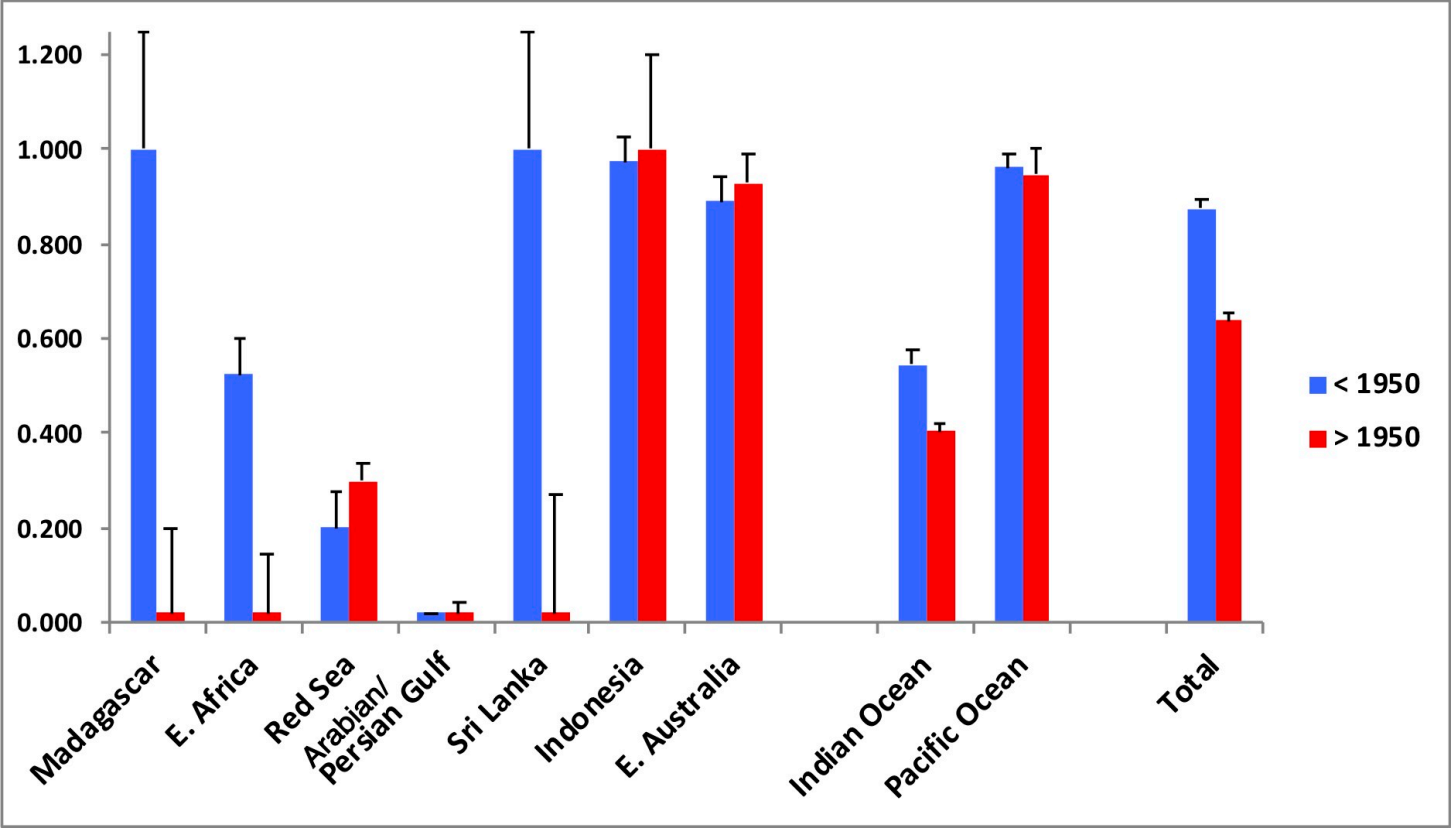
Unbiased haplotype diversities (and SEs) for samples collected before 1950 (<1950) and after 1950 (>1950). Sample sizes are listed in S2 Table.

The overall genetic diversities within the population samples from Indonesia and Australia were similar to those previously reported from those populations [15]. However, both haplotype and nucleotide diversities within the Western Indian Ocean population samples (mean H = 0.20, mean π = 0.17%) were considerably lower than within our Indonesian and Australian samples (mean H = 0.90, mean π = 3.2%) (S2 Table). Eighty-seven specimens with location information also had information on their date of collection. When these specimens were divided into two approximately equal sample groups by date of collection (those collected before or after 1950), it was clear that some of those collected prior to 1950 had significantly higher genetic diversity (S2 Table). This was most apparent in the measure of unbiased haplotype diversity (Fig 6), which was higher overall in the pre-1950 samples, and particularly so in three of the five Indian Ocean regions (namely Madagascar, East Africa and Sri Lanka), although within-region sample sizes were low.

The samples were also analysed for evidence of significant changes in population size over both short and long time-frames, using a range of parameter estimates (detailed in S2 Table). Our analyses provided some evidence of an evolutionarily recent expanding population size, from the significantly negative values of Tajima’s D and Fu’s Fs, particularly for the East African and Red Sea samples (S2 Table). However, Bayesian skyline analyses did not reveal any significant increases in population size over evolutionary time within any region (results not shown). There was some evidence of a change in effective population size over recent ecological time, in the estimates of θ (2Nu) estimated from π (nucleotide diversity). Our overall estimate of θ reduced by half in all specimens collected before compared to after 1950 (S2 Table). Dramatic reductions in θ from before to after 1950 were also observed in the samples from Madagascar, East Africa and Sri Lanka.

## Discussion

The phylogeographic analysis of historical samples from the entire range of the dugong indicates the presence of significant genetic variation and geographical structuring. Three completely new and divergent mtDNA lineages were found among the samples from the Indian Ocean region, namely the Sri Lankan, the Western Indian Ocean (combining East Africa, the Red Sea and the Arabian/Persian Gulf) and the Madagascan lineages. These are distinct from all of the previously described lineages from the eastern part of the species’ range [15] and therefore deserve further investigation (with nuclear DNA markers) as potentially distinct evolutionarily significant units (ESU’s) [55]. In particular, the unique haplotype lineage found in specimens from Madagascar indicates that any remaining animals around Madagascar should be considered for a special conservation status as they represent a genetically unique and completely isolated dugong population. Given the comparative genetic uniformity among dugong populations from the remainder of the Western Indian Ocean region, even between currently presumably isolated populations in the Red Sea and the Arabian/ Persian Gulf, it was not expected that the relatively short distance across the Mozambique Channel would act as such a strong barrier to migration in this species.

Little geographical structuring is detectable among the other populations in the Western Indian Ocean region. This may not reflect the true current state of connectivity among these somewhat isolated populations and may simply be the result of low detectable genetic variation. Further analyses of additional, longer mtDNA fragments are likely to reveal additional variation that would prove useful in better determining the degree of isolation of these populations.

In particular, the population off Mozambique, while comprising the same mtDNA haplotype as other localities in the WIO region, such as Kenya and Tanzania, should also be considered for a special conservation status, as it presents (to our knowledge) one of the few remaining viable populations (of about 250 animals) in the WIO region. As such it may represent a valuable ‘source population’ for the region.

The South Indian/ Sri Lankan population, although not unique genetically, does contain a unique mtDNA lineage, and is genetically divergent from all other populations, enhancing its conservation status. This population appears to be genetically intermediate between the WIO and Indonesian/Australian populations, a pattern that has also been observed in two species of dolphin sampled from the Bay of Bengal [56].

### Evolutionary history

Unfortunately, there are no reliable estimates of the rate of evolution within the dugong (or Sirenian) lineage. Previous estimates for the mtDNA control region (D-loop in vertebrates) have ranged from a “standard” mtDNA rate of 1% per MY to 28% per MY [15, 57, 58]. The upper estimate was based on an assumption of the divergence of sister “Widespread” and “Restricted” lineages of Australian dugong being driven by the most recent emergence of a Torres Strait land-bridge (between Australia and New Guinea) at around 115 KYA. We suspect that these lineages diverged well before this time, particularly as our analysis has shown that they are not sister lineages, and thus the true evolutionary rate for this locus is likely to be much lower. In the absence of reliable rate estimates, we require a divergence dating analysis that incorporates reliable estimates of divergences among closely related species. This information now exists thanks to the comprehensive analysis of fossil, morphological and molecular data of the Sirenian lineage provided by Springer et al [54], although it is necessary to go back more than 20 MY to the nearest species divergence (from Steller’s sea cow, *H*. *gigas*). Our new mtDNA control region sequence from the extinct Steller’s sea cow enabled a robust dated phylogenetic analysis of the Sirenian lineage.

Our Bayesian estimation of the times of divergence among dugong lineages should be a reliable measure of the relative ages of the dugong lineages. However, the specific age estimates should be regarded more cautiously. Although excellent for dating within-species divergences, the mtDNA control region is not the best locus to use for dating divergences back to the ancestors included in this analysis (> 20MY), due to its rapid rate of evolution, relatively unconstrained pattern of evolution, and potentially rapid approach to saturation [59]. Another locus, such as cytochrome b, is more likely to behave in a clock-like fashion over the time frame addressed here. Unfortunately, we were unable to successfully amplify and sequence sufficient cytochrome b fragments from our historical samples for this purpose. In the absence of a slower evolving locus, we consider this analysis of the mtDNA control region provides our best estimate yet of the likely ages of divergence within dugong. However, we suspect that these are over-estimates due to saturation in base substitution, despite the allowances made within our relaxed model of evolution for variable rates over time [60]. We are much more confident in our estimation of the relative order of divergences.

The dated phylogeny (Fig 3) suggests that the Indian Ocean dugong were only recently derived from dugong from Australia and the Western Pacific. In addition, the great bulk of the genetic diversity in the species resides within the Indo-Australian archipelago, a pattern seen commonly in a number of broadly-distributed Indo-Pacific marine species [47]. The diversity found within the Indian Ocean is only a fraction of that seen further east and appears to have been derived relatively recently in the species’ evolutionary history (Fig 3). The phylogenetic order of divergence dates of the various dugong mtDNA lineages indicates that the Australian “Widespread” and “Restricted” lineages are not in fact recent sister clades, as proposed by Blair et al [15]. Instead, they are simply two of a range of deeply diverged lineages within the species that may have diverged 10 or more MYA. Blair et al [15] suggested that the Torres Strait Pleistocene land-bridge (between Australia and New Guinea) drove the divergence between these two lineages. Instead, it appears more likely that the recurrent existence of the land-bridge during the last several hundred thousand years isolated the east Australian coast repeatedly, resulting in one lineage becoming peripherally isolated along that coast (the “Restricted” lineage). At the western extreme of the species’ distribution, a similar phenomenon appears to have driven the recent divergence of the Madagascar lineage. This pattern of peripherally isolated, lower diversity mtDNA lineages at the extremes of the species’ distribution, and higher diversity, more strongly connected populations in the centre of the current distribution, matches the predictions of the core-periphery hypothesis [61]. Several broadly-distributed Indo-Pacific marine species have now been shown to exhibit this pattern [47, 62].

Of note is the relatively old age of the major dugong lineages, which appear to be of the same order of magnitude as the age of manatee and elephant species (Fig 3). This suggests that dugongs have inhabited at least the Indo-Australian region for a considerable period of time, but that there has been sufficient mixing of populations over that period to prevent species-level divergence, unlike in manatees. The evidence suggests dugong colonized the WIO more recently. The broad and very patchy distribution of dugong along the WIO coast is unlikely to be supporting extensive gene flow. However, there must have obviously been sufficient gene flow in the recent past to prevent major genetic divergence of populations, apart from Madagascar.

### Genetic and demographic changes over time

This dataset offers the opportunity to examine genetic and demographic changes over both a long, evolutionary timescale (from the coalescent relationships among mtDNA haplotypes) and a short, ecological timescale (from the wide historical collection dates of the samples: 1827 to 1996). Previous coalescent analyses of Australian samples of dugong had provided evidence of recent significant increases in effective population size within at least the “Widespread” Australian lineage over the last few thousand years [15]. Our analyses provided some evidence of recently expanding populations in the WIO from summary statistics (significantly negative Tajima’s D and Fu’s Fs values). However, comprehensive coalescent skyline analyses on both local and regional populations did not reveal evidence of significant demographic change.

On the ecological timescale, there was some evidence of a drop in genetic diversity in a number of locations from the older samples (collected before 1950) compared to the more recent samples (collected after 1950), which may reflect an accompanying drop in dugong population sizes. This certainly matches the known reports of major population reductions since the 1960s [2, 5]. Given the relatively low sample sizes when divided into temporal population categories, we emphasize that the fine-scale results should be interpreted with caution. Nevertheless, they do indicate a reduction in diversity in many of the Indian Ocean populations sampled, which remains when the Indian Ocean samples are pooled, and deserves further investigation.

### Conclusion

This study has revealed unique, previously unidentified lineages of dugong present in the Indian Ocean, and suggests that valuable genetic diversity has already been lost in this region over the past 150 years. The phylogeographic analyses of our historical samples indicate low levels of gene flow between most biogeographic regions, highlighting in particular the isolation of dugongs from Madagascar and the Comores. In contrast, there is little genetic diversity or population divergence along the rest of the Western Indian Ocean coastline.

It is expected that the *ex-situ* sampling approach employed here and the resulting data will contribute towards a more comprehensive population genetic comparison of the dugong throughout its entire range. Such an analysis awaits the imminent publication by other research groups of several additional mtDNA data sets from newly-sampled regions in the Western Pacific. Together with on-going analyses of nuclear DNA markers in many of these samples, the analysis will contribute to a regional assessment of the dugong's conservation status. This in turn would assist in the design and implementation of sound and effective *in-situ* conservation and management strategies, aiding in the successful conservation and management of the species. Further genetic analyses of historical and contemporary dugong samples are likely to reveal additional insights into the conservation status of the Western Indian Ocean populations, including their rate of loss of genetic diversity. In this respect, further efforts to locate additional material, particularly from contemporary animals (stranded, bycaught, hunted etc.) would assist in a more detailed assessment of population structure and connectivity in this region.

## Supporting information

S1 Table Bayesian estimates of age of Most Recent Common Ancestor (MRCA) of identifiable mtDNA lineages. Data are based on Beast analysis using prior divergence dates of outgroup species (see Fig 3). (HPD–highest posterior density; MYA—million years ago).
(PDF)

S2 Table Diversity estimates and measures of departure from neutrality for each dugong region sampled. All samples within each region are presented first, followed by samples within each region divided into collection periods before 1950 (<1950, with pop. suffix -1) and after 1950 (>1950, with pop. suffix-2). Samples without collection date are excluded from the dated population samples.
(PDF)

S3 Table Details for provenance of samples. (PDF)

S1 Fig Phylogenetic tree of relationships among 355bp mtDNA control region haplotypes from all samples. Previously known and new lineages are indicated. Collection locations are indicated by colour where known. Support values for all major lineages are shown (neighbor-joining bootstrap / maximum likelihood bootstrap / Bayesian posterior probability). A reference Genbank sequence (EU835761) is included. All previous Genbank sequences from the Western Pacific fell into the lineages reported in Blair *et al*. 2014.
(PDF)

S1 Protocol Quality control for DNA extraction, PCR amplification and sequence assembly. (PDF)
