# Supplementary material for: Phylogeography of the dugong (Dugong dugon) based on historical samples identifies vulnerable Indian Ocean populations
Source: PLoS One. 2019 Sep 11;14(9):e0219350. doi: 10.1371/journal.pone.0219350 (PMC6738584; doi:10.1371/journal.pone.0219350)
Supplement: S1 Table — Data are based on Beast analysis using prior divergence dates of outgroup species (see Fig 3). (HPD–highest posterior density; MYA—million years ago). (PDF) [file pone.0219350.s001.pdf]

Clade	MRCA mean (MYA)	MRCA HPD interval
“Sri Lankan” (SL)	1.88	0.05 - 5.90
Madagascar/Comores	4.10	0.66 – 10.87
WIO	4.29	1.12 - 10.16
WIO+SL	6.33	1.76 - 13.44
WIO+SL+Madagascar/Comores	10.04	4.00 – 18.20
“Restricted” Australian	3.62	0.13 – 11.53
“Widespread” Australian	7.06	1.69 – 14.79
“Eastern”	9.10	2.48 – 17.70
All dugong	19.08	11.84 – 25.75
