# Supplementary material for: Phylogeography of the dugong (Dugong dugon) based on historical samples identifies vulnerable Indian Ocean populations
Source: PLoS One. 2019 Sep 11;14(9):e0219350. doi: 10.1371/journal.pone.0219350 (PMC6738584; doi:10.1371/journal.pone.0219350)
Supplement: S2 Table — All samples within each region are presented first, followed by samples within each region divided into collection periods before 1950 (<1950, with pop. suffix -1) and after 1950 (>1950, with pop. suffix-2). Samples without collection date are excluded from the dated population samples. (PDF) [file pone.0219350.s002.pdf]

Pop	N	Na	Ne	I	H	uH	pi (%)	theta(pi)	Taj D	Fu Fs
**Mad**	6	2	1.38	0.45	0.28	0.33	0.23	0.67	-1.13	-8.09*
**EAfr**	16	3	1.29	0.46	0.23	0.24	0.13	0.38	-1.70*	-30.00*
**RedS**	20	4	1.37	0.59	0.27	0.28	0.30	0.87	-1.55*	-34.00*
**PersG**	17	1	1.00	0.00	0.00	0.00	0.00	0.00	0.00	24.00
**SLan**	5	3	2.78	1.05	0.64	0.80	2.39	6.80	-0.84	-0.87
**Indon**	16	13	11.64	2.51	0.91	0.98	3.42	9.68	0.61	-8.56*
**EAus**	20	11	9.09	2.29	0.89	0.94	3.21	9.10	0.94	-13.25*
**Indian Ocean**	58	11	1.78	1.10	0.43	0.43	0.60	1.78	-2.16*	-4.91*
**Pacific Ocean**	29	19	15.86	2.85	0.93	0.97	3.49	10.42	0.63	-4.13
**Mad1**[1]	2	2	2.00	0.69	0.50	1.00	0.68	2.00	0.00	0.69
**Mad2**[2]	4	1	1.00	0.00	0.00	0.00	0.00	0.00	0.00	30.00
**EAfr1**	7	3	1.81	0.80	0.45	0.52	0.29	0.86	-1.36	-9.22*
**EAfr2**	5	1	1.00	0.00	0.00	0.00	0.00	0.00	0.00	14.00
**RedS1**	7	1	1.00	0.00	0.00	0.00	0.00	0.00	0.00	34.00
**RedS2**	12	4	1.71	0.84	0.42	0.45	0.48	1.41	-1.11	-17.03*
**PersG1**	1	1	1.00	0.00	0.00	0.00	0.00	0.00	0.00	-
**PersG2**	16	1	1.00	0.00	0.00	0.00	0.00	0.00	0.00	30.00
**SLan1**	2	2	2.00	0.69	0.50	1.00	5.68	16.00	0.00	2.77
**SLan2**	2	1	1.00	0.00	0.00	0.00	0.00	0.00	0.00	24.00
**Indon1**	9	8	7.36	2.04	0.86	0.97	1.71	4.94	-1.02	-4.92
**Indon2**	3	3	3.00	1.10	0.67	1.00	4.52	12.67	0.00	1.39
**EAus1**	9	6	4.76	1.68	0.79	0.89	2.54	7.22	0.16	-3.69*
**EAus2**	8	6	5.33	1.73	0.81	0.93	2.01	5.79	-0.86	-3.50*
**Indian Ocean1**	19	7	2.06	1.18	0.51	0.54	1.02	3.05	-2.14*	-0.14
**Indian Ocean2**	39	6	1.64	0.87	0.39	0.40	0.43	1.30	-1.36	-0.56
**Pacific Ocean1**	18	13	10.8	2.47	0.90	0.96	3.29	9.85	1.16	-1.83
**Pacific Ocean2**	11	8	7.11	2.02	0.86	0.94	3.08	9.21	-0.03	-0.04
**<1950**	37	20	6.68	2.51	0.85	0.87	3.18	9.06	0.18	-24.77*
**>1950**	50	14	2.66	1.65	0.62	0.64	1.98	5.66	-0.61	-25.36*
