# Supplementary material for: Phylogeography of the dugong (Dugong dugon) based on historical samples identifies vulnerable Indian Ocean populations
Source: PLoS One. 2019 Sep 11;14(9):e0219350. doi: 10.1371/journal.pone.0219350 (PMC6738584; doi:10.1371/journal.pone.0219350)
Supplement: S3 Table — (PDF) [file pone.0219350.s003.pdf]

Sample Code	Accession no.	Museum/University Collection	Location	Region	Collection Date	Accession Date
EJ-Indon	1996,109	Edinburgh	UAE, Abu Dhabi, Abu Al-Abiyadh	Northern Indian Ocean	-	1996
Liii PNG	ZMA 8869	Leiden	Sorong, New Guinea	Indonesia_PNG	Jan 1910	-
L49_Indon	ZMA 872	Leiden	Indian Archipelago	Indonesia_PNG	-	-
EL_IndOc	Free Church college	Edinburgh	Indian Ocean	Indian Ocean	-	-
Lvi_unkn	ZMA (2896)	Leiden	-	-	-	-
L9_Indon	ZMA 2113	Leiden	Kampong Tonga-Rotti, bij Timor	Indonesia_PNG	Nov/Dec 1954	-
Li_Indon	ZMA 23.739	Leiden	bij Mangaar, Biliton, Indonesia	Indonesia_PNG	1932-1933	-
Lo6-13_MB	1027 g	London	Moreton Bay	East Australia	-	-
EP_unkn	1890,65	Edinburgh	-	-	-	1890
EE_unkn	1996.83.4	Edinburgh	-	-	-	1996
EF_unkn	1996.83.4	Edinburgh	-	-	-	1996
EM_unkn	1996.83.6	Edinburgh	-	-	-	1996
EG_Slan	1836.1.14	Edinburgh	[Sri Lanka]	Eastern Indian Ocean	-	1836
ES_unkn	1827.27.1	Edinburgh	-	-	-	1827
L11_unkn	ZMA 25.385	Leiden	-	-	-	-
Lvii_unkn	ZMA 23.812	Leiden	-	-	-	-
Lo31-13	1991,405	London	Magnetic Island	East Australia	-	1991
Lo32-13-Qld	1991,412	London	Picnic Bay	East Australia	31 Jul. 1965	1991
Lo7-13 NE coast Australia	1027 c, 1846.7.7.19	London	NE coast of Australia	East Australia	-	1846
EN_unkn	1891.55.10	Edinburgh	-	-	-	1891
Lo1991.407_Qld	1991,407	London	Picnic Bay, Australia	East Australia	-	1991
Lo23_unkn	1957.8.13.3	London	-	-	13 Aug. 1957	1957
Lo26-13-Qld	1991,415	London	Radical Bay, Australia	East Australia	-	1991
Lo1991.406_Qld	1991,406	London	Picnic Bay, Australia	East Australia	-	1991
Lo24-Jor	1946.8.6.4	London	Aqaba, Red Sea	Northern Indian Ocean		1946
V58 Qld	2838	Vienna	Queensland	East Australia	1892-1893	-
Lo13-13_unkn	1027o, 1862.2.8.4	London	-	-	-	1862
M5_PNG	1913/1533	Munich	Neupommern	Indonesia_PNG	-	1913
Lo9-13_NicobarIsl.	1888.3.20.23	London	Nicobar Islands	Eastern Indian Ocean	-	1888
Lii_Indon	ZMA 871	Leiden	Indian Archipelago	Eastern Indian Ocean	-	-
Lo3-13_Moreton Bay	1027 i	London	Moreton Bay	East Australia	-	-
Lo1-13-TS	1027M, Reg. No. 1882.1.26.11	London	Hammond Island, Torres Straits	Northern Australia	1882	-
M2_PNG-s	1913/511	Munich	Blanchebucht, Neupommern, New Britain	Indonesia_PNG	12 Aug. 1913	1913
M9_PNG	1913/510	Munich	Blanchebucht, Neupommern, New Britain	Indonesia_PNG	12 Aug. 1913	1913
Lo5-13-TS	1027 e, 1846.8.26.4	London	Darnley Island, Torres Straights	Northern Australia	-	1846

Lo1991.409_Qld	1991,409	London	Picnic Bay, Australia	East Australia	-	1991
Lo28-13	1991,43	London	St. Paul's Mission/Torres Strait	Northern Australia	-	1991
Lo24-13	1991,425	London	Yarrabah, Australia	East Australia	-	1991
Lo29-13	1991,428	London	Australia	Australia	-	1991
V56_IndOc	7548	Vienna	Indian Ocean	Indian Ocean	1876	-
Lo42_Moz-S	1917.6.12.1	London	Delagoa Bay, Mozambique	Western Indian Ocean	12. June 1917	1917
Br23-unkn-s	unregistered	Bremen	-	-	-	-
Br 28_PNG	3265	Bremen	Papua New Guinea	Indonesia_PNG	1912/13	-
Br27_unkn	2691	Bremen	-	-	-	-
Br34_unkn	2688	Bremen	-	-	-	-
Br32_unkn	4115?	Bremen	-	-	-	-
Br 29_PNG	3264	Bremen	Papua New Guinea	Indonesia_PNG	1912/13	-
Br_25	4114?	Bremen	-	-	-	-
Br_26	2689	Bremen	-	-	-	-
Br_33	2687	Bremen	-	-	-	-
Br_31	2690	Bremen	-	-	-	-
Lo8-13_Moreton Bay	1027 f	London	Moreton Bay	East Australia	-	-
Pr_1047_unkn	1047	Prague	-	-	-	-
Lo1848.8.29.7_MB	1848.8.29.7	London	Morton Bay, Australia	East Australia	-	1848
Br30_unkn	4113	Bremen	-	-	-	-
Lo1933.3.2.1 Qld	1933.3.2.1	London	Queensland, Australia	East Australia	-	1933
V55_Qld	St. 291	Vienna	Queensland	East Australia	15 May 1901	-
Lo43_unkn	1027 h	London	-	-	-	-
Lo20-13_Qld	1933.3.2.2.	London	East coast of Queensland	East Australia	1933	1933
Lo11-13-Aus	1027d	London	Australia	Australia	-	-
EK_unkn	1886.16.4	Edinburgh	-	-	-	1886
Lo23-13_unkn	1027a, 1852.6.26.1	London	-	-	-	1852
L10_unkn	ZMA 2896	Naturalis	-	-	-	-
Pr_OM37_Qld	OM-37	Prague	Queensland, Australia	-	-	-
Lo27_unkn	unregistered	London	-	-	-	-
Br24_unkn	unregistered	Bremen	-	-	-	-
Lo10-13_unkn	1027 L, 1882.1.26.10	London	-	-	-	1882
Pr_OM175_unkn-s	OM-175	Prague	-	-	-	-
Lo4-13_QLd-m	1027 b, 1845.7.5.25	London	NE coast of Australia	East Australia	1845	1845
Lo18-13_Queensland	1957.8.13.2	London	Queensland	East Australia	-	1957
EO_unkn	1886.16.3	Edinburgh	-	-	-	1886
Lo26_NZ	unregistered	London	Chatham Islands, New Zealand	NZ	-	-
Lo25-13_SA-m	1857.10.24.74	London	South Australia	Australia	-	1857
Lo21_Red Sea	unregistered	London	Red Sea	Northern Indian Ocean	-	-

Lo41 Qld	1885.2.21.1 1027.n.	London	Repulse Bay, N. Australia	Northern Australia	21 Feb. 1885	1885
Lo22-13_SLan-sp	1946.8.6.1	London	Ceylon	Eastern Indian Ocean	-	1946
PE_H_Mad	N2362	Port Elizabeth	Madagascar	Western Indian Ocean	Apr. 1994	-
P10_Com	1938-535	Paris	Comores	Western Indian Ocean	-	1938
L48_unkn	unregistered	Leiden	-	-	-	-
L47_Mad	ZMA 8865	Leiden	Ankiny, bij Ampanpamena monding van de Anbazaona Nrd. Madagascar	Western Indian Ocean	3 Jan. 1964	-
P11 Mad	1938-534	Paris	Befotaka, Nosy Be, Madagascar	Western Indian Ocean	1929	1938
L45_Mad	6423 a + b	Leiden	Ankiny, Monding Id, Anbazaona, Madagascar	Western Indian Ocean	3 Jan. 1964	-
L46_Mad	ZMA 6428	Leiden	Ankiny, bij Ampanpamena monding van de Anbazaona Nrd. Madagascar	Western Indian Ocean	3 Jan. 1964	-
PE_E_unkn	N2639	Port Elizabeth	-	-	-	-
B69313_Egy	69313	Berlin	Koseir, Egypt	Northern Indian Ocean	-	-
PE_B_UAE		Port Elizabeth	Murawah Island, UAE	Northern Indian Ocean	Aug. 1995	-
M8_Tan	1914/710	Munich	Insel Mafia, Tanzania	Western Indian Ocean	-	1914
Lo35_AbuDhabi UAE	2005,52	London	Abu Dhabi, UAE	Northern Indian Ocean	29 May 1905	2005
Lo36_Abu Dhabi UAE	2005,51	London	Abu Dhabi, UAE	Northern Indian Ocean	14 Feb. 1977	2005
Lo31-Bah	1966.9.7.1	London	caught 4m off Askar, Bahrain	Northern Indian Ocean	7 Sep. 1966	-
Lo40_unkn	1870.8.16.1	London	-	-	16 Aug. 1870	-
Lo34_UAE	2005,102	London	East of Ras al Qila, UAE	-	15 Mar. 1996	2005
MA755_Egy	ZSRO MA 755	Rostock	Hurghada, Red Sea	Northern Indian Ocean	10 to 24 Sep. 1994	
Lo37_UAE	2005,5	London	Abu Dhabi, UAE	Northern Indian Ocean	14 Feb. 1977	-
HH_unkn	no number	Hamburg	-	-	-	-
P13_Tan	1901-711	Paris	Tanzania, btw. Mombasa & Zanzibar	Western Indian Ocean	1901	1901
Lo22_unkn	1950.1.24.1	London	-	-	24 Jan. 1950	1950
S11_Egy	1288 1/2	Stuttgart	Kosseir, Red Sea	Northern Indian Ocean	1869	-
V51_Egy	610	Vienna	Dahab, Red Sea	Northern Indian Ocean	4 Apr. 1896	-
L50_UAE	ZMA 24.584	Leiden	Abu Dhabi, Persian Gulf	Northern Indian Ocean	Jan. 1993	-
S22_RedSea-s	31793	Stuttgart	Red Sea	Northern Indian Ocean	1870	-
M10_unkn	1939/130	Munich	-	-	-	1939
Lo17_Mafia Tanganyika	1932.11.4.3	London	Mafia Id, Tanganyika	Western Indian Ocean	4 Nov. 1932	1932
P1_Djib	1981-153	Paris	Loyada, Djibouti	Northern Indian Ocean	1 Dec. 1980	1981

S14_unkn	31800	Stuttgart	-	-	-	-
S13_unkn	31794	Stuttgart	-	-	-	-
Lo16_Yem	1957.8.13.4	London	Loheiya, Red Sea, Arabia	Northern Indian Ocean	13 Aug. 1957	1957
P8_Djib	1981-6	Paris	Djibouti	Northern Indian Ocean	2 Dec. 1979	1981
B69309	69309	Berlin	New Ireland	Indonesia_PNG	-	-
S12_Sud	36892	Stuttgart	Suakin, N. Sudan	Northern Indian Ocean	1982	-
EB_Slan	1949,11	Edinburgh	Manaar, off Ceylon	Eastern Indian Ocean	-	1949
Lo15-13-Slan	1953.11.20.1	London	Palk Strait	Eastern Indian Ocean	-	1953
Lo29_unkn	1953.11.20.2	London	-	-	20 Nov. 1953	1953
Lo38_Slan	1946.8.6.2	London	Ceylon	Eastern Indian Ocean	6 Aug. 1946	1946
EQ_unkn	1890,65	Edinburgh	-	-	-	1890
Lo14-Manaar india	371a	London	Straits of Manaar, India	Eastern Indian Ocean	-	-
Pr_9891	9891	Prague	-	-	-	-
Lo28_unkn	1953.11.20.1	London	-	-	20 Nov. 1953	1953
P12_unkn	1925-14	Paris	-	-	-	1925
Lo16-13_Bah	70/1517	London	Bahrain, Persian Gulf	Northern Indian Ocean	22 Feb. 1970	1970
P5_Djib	1981-184	Paris	Loyada, Djibouti	Northern Indian Ocean	-	1981
V57_unkn	unregistered	Vienna	-	-	1914	-
P4_Djib	1981-157	Paris	Loyada, Djibouti	Northern Indian Ocean	20 Jan. 1981	1981
P3_Djib	1981-156	Paris	Loyada, Djibouti	Northern Indian Ocean	2 Apr. 1980	1981
Lo20_Tan	1932.11.4.4	London	Mafia Id, Tanganyika	Western Indian Ocean	4 Nov. 1932	1932
B69310_Egy	69310	Berlin	Koseir, Egypt	Northern Indian Ocean	-	-
Lo18a-Bah	BM70.1515 No.220	London	Bahrain, Persian Gulf	Northern Indian Ocean	29 Apr. 1970	1970
Lo19-13-Bah	70,1516	London	Bahrain, Persian Gulf	Northern Indian Ocean	14 Apr. 1969	1970
PE_C_UAE7	-	Port Elizabeth	UAE	Northern Indian Ocean	Aug. 1995	-
S15_RedSea	31797	Stuttgart	Red Sea	Northern Indian Ocean	1870	-
PE_D_UAE	-	Port Elizabeth	Sand Bank S. of Bii Tinah Island, UAE	Northern Indian Ocean	Aug. 1995	-
PEA_UAE	-	Port Elizabeth	Murawah Island, UAE	Northern Indian Ocean	Aug. 1995	-
Br35-Singapore	unregistered	Bremen	Singapore	Indonesia_PNG	1887	-
S17_Egy	18005	Stuttgart	Kosseir, Red Sea	Northern Indian Ocean	1869	-
Lo32_LamuKenya	1885.4.20.2	London	East coast of Lamu, Kenya	Western Indian Ocean	-	1885
M6a-Tan	1907/857	Munich	German Eastafrica	Western Indian Ocean	-	1907
ED_UAE	1996.110.1	Edinburgh	UAE, Abu Dhabi, Sir Bani Yas Island	Northern Indian Ocean	-	1996
Lo15_Tan	1932.11.4.2	London	Mafia Id, Tanganyika	Western Indian Ocean	4 Nov. 1932	1932
M1_unkn-s	AM530	Munich	-	-	-	-

V52_Egy-s	7545	Vienna	Dahab, Gulf of Aquaba, Sinai, Egypt	Northern Indian Ocean	-	-
HH_3539_Africa	3539	Hamburg	Eastafrica	Western Indian Ocean	Mar. 1957	-
V53_Egy	611	Vienna	Dahab, Red Sea	Northern Indian Ocean	4 Apr. 1896	-
S19_Africa	6915	Stuttgart	East Africa	Western Indian Ocean	-	-
S18_Tan	18006	Stuttgart	Mafia, German Eastafrica, Pwani Reg., Mafia Channel	Western Indian Ocean	1912	-
S16_Red Sea	31796	Stuttgart	Red Sea	Northern Indian Ocean	1870	-
PE_G_Moz	N2361	Port Elizabeth	Inhambane, Mozambique	Western Indian Ocean	Oct. 1994	-
PE_F_Moz	N2638	Port Elizabeth	Bazaruto, Mozambique	Western Indian Ocean	-	-
P7_Djib	1981-152	Paris	Waramous, Boulass, Djibouti	Northern Indian Ocean	17 Oct. 1979	1981
P6_Djib	1981-158	Paris	Loyada, Djibouti	Northern Indian Ocean	20 Jan. 1981	1981
P2_Djib	1981-155	Paris	Loyada, Djibouti	Northern Indian Ocean	1 Dec. 1980	1981
Lo44_Red Sea	1870.8.16.1 1534a	London	Red Sea	Northern Indian Ocean	-	1870
Lo25-Bah1	70,1513	London	Bahrain	Northern Indian Ocean	14 Apr. 1969	1970
Lo19_Bah	70,1516	London	Bahrain, Persian Gulf	Northern Indian Ocean	29 Apr. 1970	1970
M4_Tan	1913/80	Munich	Mafia Island, Tanzania	Western Indian Ocean	-	1913
S20_Sud	37622	Stuttgart	30km South of Port Sudan, Sudan	Northern Indian Ocean	1983	-
Lo39_unkn	1946.8.6.3	London	-	-	6 Aug. 1946	1946
S21_Egy	18004	Stuttgart	Kosseir, Red Sea	Northern Indian Ocean	1869	-
M3_Africa	1942/138	Munich	Africa	Africa	10 Sep. 1942	1942
EC_UAE	1996.110.2	Edinburgh	UAE, Abu Dhabi, Abu Al-Abiyadh	Northern Indian Ocean	-	1996
EI_UAE	1996,109	Edinburgh	UAE, Abu Dhabi, Abu Al-Abiyadh	Northern Indian Ocean	-	1996
EA_Ken	1972.7.2	Edinburgh	Shimoni, Kivale District, Kenya	Western Indian Ocean	-	1972
B9042_Tan	9042	Berlin	Mikindani, Tanzania	Western Indian Ocean	-	-
B46165_Ken	46165	Berlin	Island of Manda, near Lamu, Eastafrica	Western Indian Ocean	-	-
