# Supplementary figures and images for: Phylogeography of the dugong (Dugong dugon) based on historical samples identifies vulnerable Indian Ocean populations
Source: PLoS One. 2019 Sep 11;14(9):e0219350. doi: 10.1371/journal.pone.0219350 (PMC6738584; doi:10.1371/journal.pone.0219350)

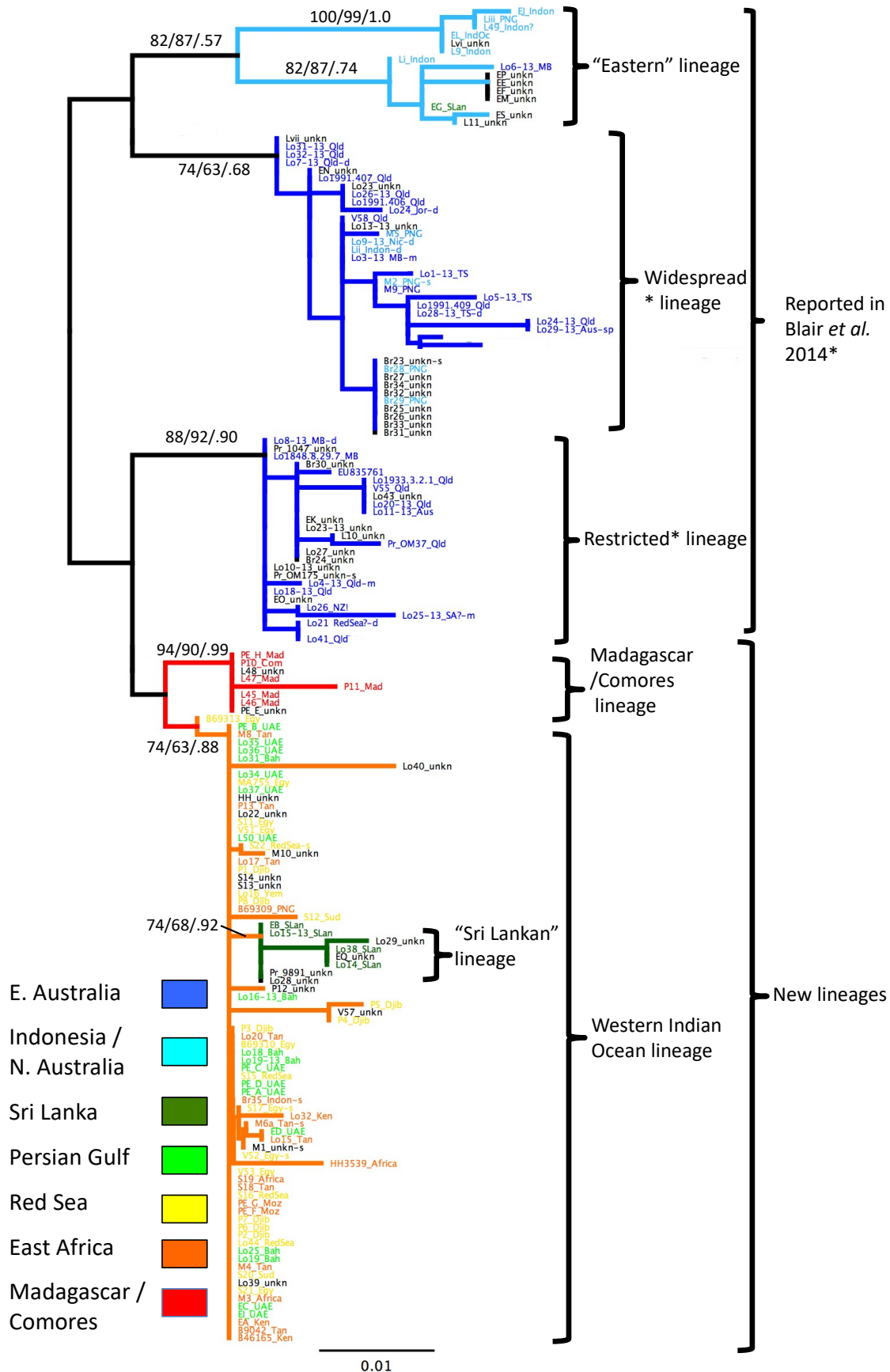

Supplement: S1 Fig — Previously known and new lineages are indicated. Collection locations are indicated by colour where known. Support values for all major lineages are shown (neighbor-joining bootstrap / maximum likelihood bootstrap / Bayesian posterior probability). A reference Genbank sequence (EU835761) is included. All previous Genbank sequences from the Western Pacific fell into the lineages reported in Blair et al. 2014. (PDF) [file pone.0219350.s004.pdf]
