# Supplementary material for: Phylogeography of the dugong (Dugong dugon) based on historical samples identifies vulnerable Indian Ocean populations
Source: PLoS One. 2019 Sep 11;14(9):e0219350. doi: 10.1371/journal.pone.0219350 (PMC6738584; doi:10.1371/journal.pone.0219350)
Supplement: S1 Protocol — (PDF) [file pone.0219350.s005.pdf]

Sampling of dugong bone and tooth/tusk material was carried out with a hand-held drill and a 1.5mm drill bit. For the purpose of aDNA analysis, 0.05g (50mg) of bone or tooth powder was sufficient for extraction. Drill bits were changed between individual samples and a new 1.5mm drillbit was used for each sample. An effort was made not to drill more than 5 or 6 samples in the same location to avoid cross-contamination between samples.
The primary laboratory analysis was conducted in a laboratory isolated from any mammal DNA and only equipment and reagents dedicated for the sole purpose of this project were used. Custom-designed primers were employed to obtain overlapping as well as forward and reverse sequences for each sample. Each sequence originated from a separate PCR reaction to ensure that sequence artefacts were not included. A number of sequences were produced from replicate DNA extractions from the same individual to help ensure that no sequence artefacts were included.
A contig of overlapping sequences was aligned for each individual, ensuring that replicate samples yielded identical sequencing results. In addition, multiple sequences obtained from multiple PCR products were identical for each individual, indicating no evidence of artefacts or contamination. Furthermore, all sequences of known geographic origin were compared to existing reference sequences from the same geographic region, where available, to ascertain that they were geographically reliable.
